# Ecolabels and the Healthfulness and Carbon Footprint of Restaurant Meal Selections: A Randomized Clinical Trial

**DOI:** 10.1001/jamanetworkopen.2025.24773

**Published:** 2025-08-04

**Authors:** Anna H. Grummon, Amanda B. Zeitlin, Cristina J. Y. Lee, Caroline Collis, Lauren P. Cleveland, Aviva A. Musicus, Joshua Petimar

**Affiliations:** 1 Department of Pediatrics, Stanford University School of Medicine, Palo Alto, California; 2 Department of Health Policy, Stanford University School of Medicine, Stanford, California; 3 Department of Population Medicine, Harvard Pilgrim Health Care Institute, Boston, Massachusetts; 4 Department of Nutrition, Harvard T.H. Chan School of Public Health, Boston, Massachusetts; 5 Science Department, Center for Science in the Public Interest, Washington, DC

## Abstract

**Importance:**

Restaurants are increasingly interested in capitalizing on consumer interest in environmental sustainability by marketing their products with ecolabels, which signal when foods are more environmentally sustainable. Ecolabels could improve the healthfulness of restaurant meal selections and reduce their carbon footprint, but this potential remains largely untested.

**Objective:**

To test whether displaying ecolabels on restaurant menus improves the healthfulness and reduces the carbon footprint of restaurant meal selections.

**Design, Setting, and Participants:**

This randomized clinical trial was conducted online in September to October 2024. A national sample of US adults (aged ≥18 years) was recruited. Participants were randomly assigned to the ecolabel or control arm. Participants viewed a restaurant menu mimicking a popular full-service restaurant and selected items they wanted to order. Statistical analyses were based on the intention-to-treat principle.

**Interventions:**

In the ecolabel arm, participants viewed a menu that displayed ecolabels next to entrées and appetizers with a lower carbon footprint (ie, below the median of 1.625 kg of carbon dioxide equivalent [CO_2_e] emissions per item). In the control arm, participants viewed a menu that did not display ecolabels.

**Main Outcomes and Measures:**

The outcomes included overall healthfulness (assessed using Ofcom Nutrient Profiling Model scores; range: 0-100, with higher scores indicating healthier items), nutrient content, and total carbon footprint of participants’ entrée and appetizer selections and entire orders (including beverages and desserts).

**Results:**

A total of 3147 participants completed the online trial (1560 men [50%]; mean [SD] age, 34.5 [12.5] years). Participants in the ecolabel arm did not select entrées and appetizers (average differential effect [ADE], 0.45 [95% CI, –0.18 to 1.09]; *P* = .16; Cohen *d* = 0.05) or entire orders (ADE, 0.47 [95% CI, –0.09 to 1.03]; *P* = .10; Cohen *d* = 0.06) that were statistically significantly healthier compared with the selections of participants in the control arm. Participants in the ecolabel arm selected entrées and appetizers (ADE, 0.87 [95% CI, 0.12-1.62] g; *P* = .02; Cohen *d* = 0.08) and entire orders (ADE, 0.82 [95% CI, 0.07-1.56] g; *P* = .03; Cohen *d* = 0.08) with more fiber, compared with the selections of participants in the control arm, but did not select entrées and appetizers or entire orders with statistically significantly different amounts of protein, sugar, saturated fat, or calorie content. Participants in the ecolabel arm selected entrées and appetizers (ADE, –0.78 [95% CI, –1.25 to –0.32] kg of CO_2_e emissions; *P* < .001; Cohen *d* = –0.12) and entire orders (ADE, –0.81 [95% CI, –1.27 to –0.34] kg of CO_2_e emissions; *P* < .001; Cohen *d* = –0.12) with lower carbon footprints than the selections of participants in the control arm.

**Conclusions and Relevance:**

In this randomized clinical trial, menu ecolabels reduced the carbon footprint of restaurant meal selections without worsening nutritional quality. Ecolabels could be a scalable, low-cost strategy to reduce the carbon emissions of restaurant food choices.

**Trial Registration:**

ClinicalTrials.gov Identifier: NCT06584539

## Key Points

**Question:**

Do ecolabels lead people to select foods from restaurants that are healthier or have a lower carbon footprint?

**Findings:**

In this randomized clinical trial with 3147 US adults, participants who viewed restaurant menus displaying ecolabels on more environmentally sustainable foods selected items with similar overall healthfulness and 9.2% lower carbon footprint compared with participants who viewed restaurant menus without ecolabels.

**Meaning:**

Ecolabels could be a scalable, low-cost strategy to reduce the carbon emissions of restaurant food choices.

## Teaser

This randomized clinical trial examines whether using labels to signify the environmental sustainability of restaurant foods has an effect on consumers’ meal selections.

## Introduction

US consumers are increasingly concerned about the environmental sustainability of their food choices. One in 3 US adults, for example, say that environmental sustainability is impactful in their decisions about what foods to buy.^1^ In turn, retailers seek to capitalize on consumer interest in sustainability by marketing their foods with ecolabels that signal when foods are more environmentally sustainable (eg, associated with lower carbon emissions). As of 2020, retailers had developed nearly 150 ecolabeling systems for food,^2^ and major manufacturers (eg, Unilever^3,4^), grocery stores (eg, Amazon Fresh^5^ and Giant Foods^6^), and restaurant chains (eg, Panera^7^ and Sweetgreen^8^) display ecolabels on their more sustainable food offerings.

Displaying ecolabels in restaurants could improve environmental sustainability at the population level given that restaurant foods account for one-third of calories consumed in the US^9^ and are a major source of carbon-intensive foods, such as red meat.^10^ It remains largely unknown, however, how ecolabels affect the carbon footprint of restaurant purchases.^11,12^ Ecolabels could also have the added benefit of leading consumers to select healthier foods, given that more environmentally sustainable foods tend to be healthier.^13,14,15,16,17,18,19,20,21^Alternatively, ecolabels might lead consumers to select less healthy foods—for example, if they generate licensing effects in which consumers who choose a “virtuous” option (eg, a food with an ecolabel) are induced to select an “indulgent” option (eg, a dessert).^22^ To our knowledge, however, only 1 study has examined the impact of ecolabels on the healthfulness of restaurant meal selections.^12^

To address these gaps, we conducted a randomized clinical trial to test whether displaying ecolabels on restaurant menus improves the healthfulness and reduces the carbon footprint of restaurant meal selections. We also examined whether younger adults^23^ (aged 18-29 years) and adults with higher interest in environmental sustainability^24^ respond differently to ecolabels.

## Methods

### Participants

From September to October 2024, the survey research panel CloudResearch Connect recruited a sample of US adults. CloudResearch uses patented technical and behavioral checks (eg, attention checks and language comprehension tests) to prevent fraudulent and duplicate responses.^25^ Individuals were eligible to participate if they were 18 years or older and lived in the US. We used quota sampling to ensure that approximately 50% of participants were younger adults (aged 18-29 years) to ensure a sufficient sample size for moderation analyses by age group. Invitations indicated that the study would involve selecting items to order from a restaurant menu. The Harvard Pilgrim Health Care Institute Institutional Review Board approved the study. The trial protocol was prospectively registered and is available in Supplement 1. All participants provided electronic informed consent. We followed the Consolidated Standards of Reporting Trials (CONSORT) reporting guideline.^26^

### Procedures

Participants completed an online experiment programmed in Qualtrics. After providing informed consent, participants were randomly assigned by Qualtrics, using a simple allocation ratio, to 1 of 2 trial arms: ecolabel or control. Participants viewed a restaurant menu designed to mimic the online ordering platform of a popular full-service restaurant in the US, similar to prior studies.^12,27,28,29^ The menu displayed 37 items, including 4 appetizers, 22 entrées, 3 desserts, and 8 beverages (eTable 1 in Supplement 2). All items were displayed on 1 screen. We aimed to include a variety of types of items (eg, entrées included bowls, burgers, sandwiches, fajitas, and salads) and to represent a range of healthfulness and carbon footprints within each type of item (eg, the burger category included 2 beef burgers, a chicken sandwich, and a vegetarian burger), but we did not apply other criteria for selecting items. The menu displayed each item with a photo, a short description (eg, Cajun Shrimp Pasta), and calorie information, but no prices were shown (eMethods in Supplement 2).

**Figure 1.**
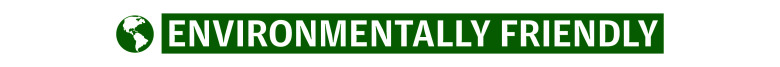
Ecolabel Displayed on the Restaurant Menu Next to More Sustainable Menu Items

In the ecolabel arm, the restaurant menu displayed ecolabels next to more environmentally sustainable items (Figure 1 depicts the ecolabel). We designed this ecolabel based on prior research indicating that labels with icons and text are more effective than icon-only or text-only labels^28,30,31^ and on a pilot experiment demonstrating that ecolabels with an earth icon and the text “environmentally friendly” are especially promising. We displayed the ecolabels beneath more environmentally sustainable entrées and appetizers (but not beverages or desserts) to match the types of foods that typically receive ecolabels at actual restaurants. We defined more environmentally sustainable items based on their carbon footprints—the greenhouse gas emissions associated with producing each food or beverage (eMethods in Supplement 2 describe carbon footprint calculations). Entrées and appetizers were eligible for ecolabels if they had a carbon footprint below the median among entrées and appetizers (1.625 kg of carbon dioxide equivalent [CO_2_e] emissions per item). This definition ensured that a diverse array of items received ecolabels (eg, burgers, fajitas, bowls, and salads) (eTable 1 in Supplement 2). In the control arm, the restaurant menu did not display ecolabels.

In both trial arms, participants were instructed to select the item or items they wished to order from the menu; no meal was specified. Participants were required to select at least 1 entrée or appetizer and no more than 4 total items. To encourage participants to select items they actually wished to eat, we informed them that 25 participants would be chosen at random to have their menu item selections delivered to them. At the end of the study, participants were debriefed that those chosen to receive their selections would instead receive an electronic gift card for $25 (slightly more than the cost of the most expensive menu item).

After participants selected items to order from the menu, they responded to survey questions as described in the next section. Median (IQR) completion time was 8.58 (6.52-11.93) minutes. Participants received incentives worth $2.25 from CloudResearch.

### Measures

We examined the healthfulness, carbon footprint, and nutrient content of participants’ selections. For these outcomes, we examined the effects among entrée and appetizer selections only (given that these were the only items eligible for ecolabels in the ecolabel arm) and across the entire order (ie, including beverages and desserts).

First, we examined the healthfulness of participants’ entrée and appetizer selections (primary outcome) and overall selections. We chose healthfulness as the primary outcome given the limited research examining the impact of ecolabels on healthfulness of food and beverage purchases.^11^ We calculated healthfulness using the UK’s Ofcom Nutrient Profiling Model scores (range: 0-100, with higher scores indicating healthier items)^32,33^; people who eat diets with healthier Ofcom scores have lower risk of obesity^34^ and cardiovascular disease.^35^ We calculated healthfulness scores for each menu item using nutrition information from the restaurant’s website and averaged these scores across all items that participants selected. Mean (SD) healthfulness scores were 59.0 (14.2) across all menu items, 64.3 (11.4) for entrées and appetizers eligible for ecolabels, and 54.3 (12.9) for entrées and appetizers ineligible for ecolabels.

Second, we examined the total carbon footprint (expressed in kg of CO_2_e emissions) of participants’ entrée and appetizer selections and their entire orders. We estimated the carbon footprint for each menu item by matching items to a database of the environmental impacts of food^36,37^ (eMethods in Supplement 2). We summed across all items participants selected to calculate total carbon footprints.

Third, we examined the nutrient content (eg, fiber, protein, and sodium) of participants’ entrée and appetizer selections and their entire orders. To provide additional context about participants’ orders and assess whether ecolabels led to licensing effects (ie, selection of an indulgent option to offset selection of a virtuous option), we also examined the total number of entrées and appetizers, beverages, and desserts ordered as exploratory (not prespecified) secondary outcomes.

Fourth, we assessed psychological outcomes that research indicates are mechanisms through which food labels influence behavior^38,39,40,41^ using measures adapted from prior studies. These outcomes included noticing the ecolabels^12^; thinking about sustainability, healthfulness, and taste while selecting items^27,42^; and perceptions of sustainability, healthfulness, and tastiness of sustainable and unsustainable menu items (defined as entrees and appetizers that were eligible and ineligible for the ecolabels, respectively).^28,42,43^ For the perception questions, participants were shown 1 sustainable item and 1 unsustainable item, both selected at random. Noticing was assessed with binary (yes or no) response options; thinking and perception questions used response scales ranging from 1 (low) to 5 (high).

Fifth, we assessed the acceptability of the ecolabels (eg, liking of ecolabels^44^). Sixth, we assessed demographic characteristics (eg, age, educational level, and annual household income) and interest in sustainability (assessed using the GREEN scale,^24^ whereby participants rated items on a scale of 1 [strongly disagree] to 5 [strongly agree]). Race and ethnicity were self-reported by participants (from the following options: American Indian or Alaska Native; Asian; Black or African American; Hispanic, Latino, or Spanish origin; Middle Eastern or North African; Native Hawaiian or Other Pacific Islander; White; or other [specified by participants]). Race and ethnicity were collected in this study to characterize the sample and compare sample characteristics to those of the US population. Survey items and response options are shown in eTable 2 in Supplement 2.

### Statistical Analysis

Using G*Power version 3.1,^45^ we estimated that the target sample size of 3100 would provide 90% power to detect a standardized mean difference between the ecolabel arm and the control arm, with a Cohen *d* = 0.12 or larger, based on prior studies of environmental^2,3,4^ and health^5,6,7^ labels. First, we used ordinary least squares regression to examine the effect of ecolabels on the primary outcome of healthfulness of entrée and appetizer selections. We regressed healthfulness on an indicator variable for trial arm (ecolabel vs control). We converted effects to standardized mean differences (Cohen *d*) to interpret whether effects were small (Cohen *d* = 0.20), medium (Cohen *d* = 0.50), or large (Cohen *d* = 0.80).^46^ Second, we examined whether the effects of the ecolabels on the primary outcome were moderated by age (younger adult [aged 18-29 years] vs older adult [aged ≥30 years]) or by interest in sustainability (GREEN scale score,^24^ treated continuously) by adding the moderator and interaction between the moderator and trial arm to the primary model. We examined age and interest in environmental sustainability as potential moderators because research suggests that younger adults and those with higher interest in environmental sustainability may be more likely to change their behavior in response to ecolabels.^23,24^ Third, we used a similar approach to assess the effects of the ecolabels on the secondary outcomes, using linear models for continuous outcomes and logistic models for binary outcomes. Fourth, we calculated the proportion of respondents who reported liking the ecolabel, wanting to see ecolabels on restaurant menus, perceiving ecolabels to be helpful, and perceiving ecolabels as increasing their control over making sustainable eating decisions.

We used Stata MP, version 18 (StataCorp LLC) for the analyses. Analyses were based on the intention-to-treat principle. We conducted 2-tailed statistical tests and considered α<.05 to be statistically significant. Missing data were minimal (0.0%-1.2%); thus, we used complete case analysis.

## Results

**Figure 2.**
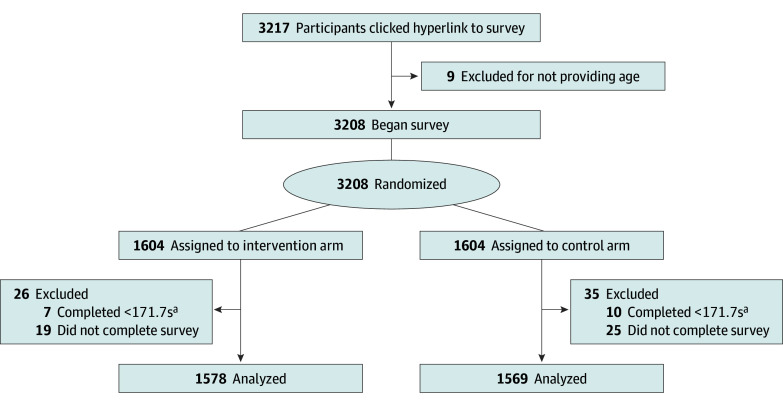
CONSORT Flow Diagram ^a^One-third of the median (IQR) completion time of 8.58 (6.52-11.93) minutes.

A total of 3147 participants were included in the analyses (Figure 2). Participants had a mean (SD) age of 34.5 (12.5) years; included 1560 men (50%), 1549 women (49%), and 11 nonbinary and other individuals (0.3%). Participants identified their race and ethnicity as follows: 12 (0.4%) American Indian or Alaska Native; 276 (9%) Asian or Native Hawaiian or Other Pacific Islander; 439 (14%) Black or African American; 294 (9%) Hispanic, Latino, or Spanish origin; 10 (0.3%) Middle Eastern or North African; 1981 (63%) White; and 108 (3%) of other race and ethnicity (Table 1). This sample was similar to the overall US population in terms of gender identity, race and ethnicity, and annual household income, but the sample included more younger adults; fewer people who identified as Hispanic, Latino, or of Spanish origin; and more people with college degrees or higher educational level (eTable 3 in Supplement 2).

**Table 1.** Participant Characteristics

Characteristic	Participants, No. (%)^a^	
	Ecolabel arm (n = 1578)	Control arm (n = 1569)
Age group, y		
18-29	782 (50)	781 (50)
30-44	479 (30)	469 (30)
45-59	229 (15)	221 (14)
≥60	88 (6)	98 (6)
Gender identity		
Women	761 (49)	788 (51)
Men	797 (51)	763 (49)
Nonbinary and other^b^	4 (0.3)	7 (0.4)
Race and ethnicity^c^		
American Indian or Alaska Native	4 (0.3)	8 (0.5)
Asian or Native Hawaiian or Other Pacific Islander^c^	149 (10)	127 (8)
Black or African American	218 (14)	221 (14)
Hispanic, Latino, or Spanish origin	134 (9)	160 (10)
Middle Eastern or North African	8 (0.5)	2 (0.1)
White	997 (64)	984 (63)
Other^d^	52 (3)	56 (4)
Educational level		
≤High school diploma or GED	218 (14)	223 (14)
Some college or technical school	291 (19)	310 (20)
Bachelor’s or associate’s degree	836 (54)	798 (51)
Graduate or professional degree	217 (14)	227 (15)
Annual household income, $		
0 to 24 999	145 (9)	188 (12)
25 000 to 49 999	333 (21)	335 (22)
50 000 to 74 999	325 (21)	348 (22)
≥75 000	755 (48)	686 (44)
Household size		
1-2	681 (44)	714 (46)
3-4	666 (43)	663 (43)
≥5	210 (13)	180 (12)
No. of children		
0	1007 (65)	1020 (66)
1-2	479 (31)	473 (30)
≥3	71 (5)	64 (4)
Political party affiliation		
Democrat	874 (56)	840 (54)
Republican	390 (25)	411 (26)
Independent	270 (17)	277 (18)
Other^e^	24 (2)	29 (2)
Interest in sustainability score, mean (SD)^f^	3.46 (0.95)	3.38 (0.91)

Abbreviation: GED, General Educational Development.

^a^ May not sum to 100% due to rounding. Missing data ranged from 0.0% to 1.0%.

^b^ Other gender identity included any identities participants self-described in a free-response box.

^c^ Race and ethnicity data were self-reported by participants in the survey. Asian and Native Hawaiian or Other Pacific Islander categories were combined due to small cell sizes.

^d^ Other race and ethnicity included multiracial and any that participants self-described in a free-response box.

^e^ Other political affiliation included any affiliation participants self-described in a free-response box.

^f^ Assessed using the GREEN scale,^24^ whereby participants rated their interest on a scale of 1 (strongly disagree) to 5 (strongly agree).

Participants in the control arm selected entrées and appetizers with a mean (SD) healthfulness score of 54.7 (9.0) on the Ofcom Nutrient Profiling Model (Table 2). The presence of ecolabels did not lead to statistically significant improvements in the overall healthfulness of entrée and appetizer selections (average differential effect [ADE], 0.45 [95% CI, –0.18 to 1.09]; *P* = .16; Cohen *d* = 0.05). The effect of ecolabels on the healthfulness of entrée and appetizer selections was not moderated by age or interest in sustainability (eg, ADE, 0.59 for younger adults vs 0.31 for older adults; *P* for interactions = .54) (eTable 4 in Supplement 2). Similar to results for entrée and appetizer selections, ecolabels did not lead to statistically significant improvements in the healthfulness of entire orders (ADE, 0.47 [95% CI, –0.09 to 1.03]; *P* = .10; Cohen *d* = 0.06).

**Table 2.** Effect of Ecolabels on Healthfulness, Carbon Footprint, Nutrient Content, and Item Composition of Restaurant Meal Selections

Outcome	Mean (SD)		Difference between ecolabel arm vs control arm		
	Ecolabel arm	Control arm	ADE (95% CI)	*P* value	Cohen *d*
**Healthfulness score** ^a^					
Entrées and appetizers	55.1 (9.2)	54.7 (9.0)	0.45 (−0.18 to 1.09)	.16	0.05
Entire order	55.1 (8.1)	54.6 (7.9)	0.47 (−0.09 to 1.03)	.10	0.06
**Carbon footprint, kg of CO_2_e emissions **					
Entrées and appetizers	7.7 (6.7)	8.5 (6.6)	−0.78 (−1.25 to −0.32)	<.001	−0.12
Entire order	8.2 (6.7)	9.0 (6.6)	−0.81 (−1.27 to −0.34)	<.001	−0.12
**Fiber, g**					
Entrées and appetizers	18.8 (10.9)	17.9 (10.4)	0.87 (0.12 to 1.62)	.02	0.08
Entire order	20.0 (10.9)	19.2 (10.5)	0.82 (0.07 to 1.56)	.03	0.08
**Protein, g**					
Entrées and appetizers	126.6 (60.0)	130.1 (60.4)	−3.50 (−7.71 to 0.71)	.10	−0.06
Entire order	131.5 (60.0)	135.3 (60.1)	−3.82 (−8.02 to 0.38)	.07	−0.06
**Sodium, mg**					
Entrées and appetizers	6126.6 (2585.1)	6096.4 (2541.5)	30.20 (−148.99 to 209.40)	.74	0.01
Entire order	6492.1 (2601.5)	6487.8 (2535.2)	4.31 (−175.25 to 183.86)	.96	0.00
**Saturated fat, g**					
Entrées and appetizers	49.5 (27.2)	50.5 (26.0)	−1.07 (−2.92 to 0.79)	.26	−0.04
Entire order	60.2 (30.2)	61.8 (28.8)	−1.64 (−3.70 to 0.43)	.12	−0.06
**Sugar, g**					
Entrées and appetizers	22.5 (12.8)	22.3 (12.6)	0.25 (−0.64 to 1.14)	.58	0.02
Entire order	75.8 (55.0)	79.6 (55.8)	−3.79 (−7.66 to 0.09)	.06	−0.07
**Calories, kcal**					
Entrées and appetizers	2574.0 (1109.1)	2586.4 (1082.1)	−12.41 (−89.00 to 64.19)	.75	−0.01
Entire order	3064.3 (1206.0)	3112.7 (1174.3)	−48.39 (−131.60 to 34.81)	.25	−0.04
**No. of items ordered**					
Entrées and appetizers	2.2 (0.8)	2.3 (0.8)	−0.02 (−0.08 to 0.04)	.53	−0.02
Beverages	0.6 (0.5)	0.6 (0.5)	−0.02 (−0.06 to 0.01)	.18	−0.05
Desserts	0.4 (0.5)	0.4 (0.5)	−0.02 (−0.06 to 0.01)	.17	−0.05

Abbreviations: ADE, average differential effect; CO_2_e, carbon dioxide equivalent.

^a^ According to the Ofcom Nutrient Profiling Model score (range from 0 to 100, with higher scores indicating healthier items).

Although ecolabels did not affect overall healthfulness of meal selections, they did lead participants to select items with lower carbon footprints. Specifically, the ecolabels led to a 9.2% reduction in total carbon footprint of entrée and appetizer selections (ADE, –0.78 [95% CI, –1.25 to –0.32] kg of CO_2_e emissions; *P* < .001; Cohen *d* = –0.12). This effect also held for entire orders (ADE, –0.81 [95% CI, –1.27 to –0.34] kg of CO_2_e emissions; *P* < .001; Cohen *d* = –0.12).

There were modest differences in the nutrient content of restaurant menu item selections between arms. Compared with selections in the control arm (without ecolabels), participants in the ecolabel arm selected entrées and appetizers (ADE, 0.87 [95% CI, 0.12-1.62] g; *P* = .02; Cohen *d* = 0.08) and entire orders (ADE, 0.82 [95% CI, 0.07-1.56] g; *P* = .03; Cohen *d* = 0.08) with 4.2% to 4.9% more fiber. There were no statistically significant differences in the protein, saturated fat, sugar, or calorie content of entrée and appetizer selections or entire orders between the ecolabel arm and control arm. However, protein was somewhat lower for both entrées and appetizer selections (ADE, –3.50 [95% CI, −7.71 to 0.71] g; *P* = .10; 2.7% less than the control arm) and entire orders (ADE, −3.82 [95% CI, −8.02 to 0.38] g; *P* = .07; 2.8% less than the control arm) and sugar was somewhat lower for entire orders (ADE, –3.79 [95% CI, −7.66 to 0.09] g; *P* = .06) in the ecolabel vs control arm. There were no differences in the number of entrées and appetizers, beverages, or desserts ordered by participants in the ecolabel arm compared with the control arm.

Participants in the ecolabel arm were more likely to report noticing ecolabels than participants in the control arm (ADE, 59 [95% CI, 56-61] percentage points; *P* < .001; Cohen *d* = 1.55) (Table 3). Compared with reactions in the control arm, the ecolabels led to more thinking about environmental sustainability (ADE, 0.57 [95% CI, 0.49-0.65]; *P* < .001; Cohen *d* = 0.49) and healthfulness (ADE, 0.20 [95% CI, 0.11-0.28]; *P* < .001; Cohen *d* = 0.16) but did not lead to differences in thinking about taste.

**Table 3.** Effect of Ecolabels on Psychological Outcomes

Outcome	Mean (SD)		Difference between ecolabel arm vs control arm		
	Ecolabel arm	Control arm	ADE (95% CI)	*P* value	Cohen *d*
Noticing ecolabels, No. (%)	1131/1575 (72)	208/1567 (13)	59 (56-61) percentage points	<.001	1.55
Thinking about sustainability	2.31 (1.2)	1.74 (1.1)	0.57 (0.49 to 0.65)	<.001	0.49
Thinking about healthfulness	2.86 (1.2)	2.66 (1.2)	0.20 (0.11 to 0.28)	<.001	0.16
Thinking about taste	4.61 (0.6)	4.65 (0.6)	−0.03 (−0.08 to 0.01)	.12	−0.06
**Perceptions of sustainable menu items**					
Perceived sustainability	3.38 (0.96)	2.90 (0.99)	0.47 (0.40 to 0.54)	<.001	0.49
Perceived healthfulness	2.96 (1.22)	2.84 (1.22)	0.12 (0.04 to 0.21)	.005	0.10
Perceived tastiness	3.62 (1.00)	3.68 (1.04)	−0.06 (−0.13 to 0.01)	.11	−0.06
**Perceptions of unsustainable menu items**					
Perceived sustainability	1.95 (1.01)	2.20 (1.03)	−0.25 (−0.32 to −0.18)	<.001	−0.25
Perceived healthfulness	2.12 (1.06)	2.18 (1.07)	−0.06 (−0.13 to 0.01)	.12	−0.06
Perceived tastiness	3.96 (1.04)	4.01 (0.99)	−0.05 (−0.12 to 0.02)	.19	−0.05

Abbreviation: ADE, average differential effect.

Compared with perceptions in the control arm, participants in the ecolabel arm perceived sustainable menu items as both healthier (ADE, 0.12 [95% CI, 0.04 to 0.21]; *P* = .005; Cohen *d* = 0.10) and more sustainable (ADE, 0.47 [95% CI, 0.40 to 0.54]; *P* < .001; Cohen *d* = 0.49) but not more or less tasty. Similarly, participants in the ecolabel arm perceived unsustainable menu items as less sustainable (ADE, −0.25 [95% CI, −0.32 to −0.18], *P* < .001; Cohen *d* = −0.25) but not more or less healthy or tasty.

Across both arms, the majority of participants (2331 of 3128 [75%]) reported that ecolabels made them feel more in control of making sustainable eating decisions (eFigure in Supplement 2). Similarly, the majority of participants reported that the ecolabels would help them choose more environmentally sustainable foods (2225 [71%]), that they liked ecolabels (2019 [65%]), and that they want to see ecolabels on restaurant menus (1981 [63%]).

## Discussion

In this randomized clinical trial with US adults, displaying ecolabels on restaurant menus next to more environmentally sustainable items did not lead to changes in overall healthfulness of participants’ selections but did lead participants to select orders with a 9.2% lower carbon footprint. The ecolabels also led participants to think more about sustainability and healthfulness without impacting how tasty participants perceived the items to be. These results suggest that ecolabels could be a promising, scalable strategy for reducing carbon emissions associated with restaurant foods without reducing the overall healthfulness or perceived tastiness of meals.

It is possible that the ecolabels tested in this study did not improve the overall healthfulness of participants’ orders because they were positively framed (ie, emphasized favorable attributes), while prior research indicates that negatively framed labels (ie, emphasized unfavorable attributes) may be more effective at influencing food purchases.^12,47,48^ One randomized clinical trial, for example, found that negatively framed high climate impact labels led participants to select healthier restaurant meals compared with control labels, while positively framed low climate impact labels did not.^12^ To maximize health benefits, retailers and policymakers could consider adopting negatively framed labels about foods’ environmental effects.

Although ecolabels did not lead to differences in the overall healthfulness of participants’ restaurant meal selections, they did lead to modest changes in nutritional content. Specifically, participants in the ecolabel arm selected items with 4% to 5% more fiber compared with participants in the control arm. These results are promising given that dietary fiber consumption reduces risk of cardiovascular disease and all-cause mortality,^49,50^ and only 4% to 6% of US children and adults meet recommendations for fiber consumption.^51,52^ We also observed some evidence that the ecolabels led participants to select items with 3% less protein compared with participants in the control arm, although these results did not reach statistical significance. This finding could be due to ecolabels leading participants to more frequently select plant-based proteins, which may have more fiber but slightly less protein than animal-based proteins. Although these results suggest ecolabels may have minor effects on protein intake, the majority of US adults meet protein recommendations.^53^ Moreover, ecolabels did not lead participants to select more desserts, suggesting that they did not generate licensing effects that induced participants to select more indulgent items. Together, our results suggest that ecolabels are unlikely to generate major unintended effects on the healthfulness of restaurant orders and may even offer modest benefits.

The ecolabels led participants to think more about environmental sustainability when they were deciding what to order and to perceive sustainable menu items as more sustainable and unsustainable items as less sustainable. Similar psychological responses to health-related labels (ie, heightened thinking about health and changes in perceptions of healthfulness) are indicative of longer-term behavior change,^38,39,40,41^ suggesting that ecolabels hold promise for encouraging consumers to eat more sustainable foods.

Although the ecolabels focused specifically on communicating environmental sustainability, their effects also spilled over to lead participants to perceive sustainable items as healthier, similar to findings in a prior study.^12^ These results suggest that ecolabels may create halo effects––that is, cause consumers to think that sustainable foods also have other positive characteristics such as being healthier. In many circumstances, these halo effects are warranted given the substantial overlap between dietary patterns that are more environmentally sustainable and those that are healthier.^13,14,15,16,17,18,19,20,21^ However, some foods that are typically viewed as more environmentally sustainable have a generally unhealthy nutritional profile (eg, meat-mimicking products tend to be high in sodium and saturated fat),^54,55^ suggesting that ecolabels could mislead consumers about the healthfulness of these products. Pairing ecolabels with health or nutrition labels could mitigate this potential unintended consequence.

### Strengths and Limitations

Strengths of this study included the randomized design, large national sample, and use of realistic stimuli mimicking a popular US restaurant chain. This study has limitations. First, we recruited a convenience sample that included more younger adults; fewer people who identified as being Hispanic, Latino, or of Spanish origin; and more people with college degrees than the overall US population. Our results therefore may not generalize to the overall population, although findings from randomized experiments conducted in convenience samples tend to replicate in national probability samples.^56,57^ Second, we examined responses to a 1-time exposure to ecolabels on a subset of menu items from a full-service restaurant shown in the context of an online experiment. The menu did not include prices. It remains unknown whether the results would generalize to repeated exposures, full menus, quick-service or other types of restaurants, in-person settings, or menus with prices. Third, we measured hypothetical selections, although we gave participants an incentive to select items they actually wished to order. Fourth, although the carbon footprints of menu items were constant across the trial arms, they were estimated and may not reflect exact preparation methods.

## Conclusions

In this randomized clinical trial with a large national sample, placing ecolabels on more environmentally sustainable menu items reduced the carbon footprint of restaurant meal selections without negatively affecting the overall healthfulness of selections. Ecolabels could be a scalable, low-cost strategy to reduce the carbon emissions associated with restaurant food choices.

## Notes

Supplement 1.
Trial Protocol

Supplement 2.
eMethods.
**eFigure.** Acceptability of Ecolabels, n = 3,147 US Adults **eTable 1.** Characteristics of Menu Items Shown in the Experiment **eTable 2.** Survey Measures Used in Experiment **eTable 3.** Comparison of Characteristics of the Study Sample (n = 3,147 US Adults) to National Estimates **eTable 4.** Impact of Ecolabels on Healthfulness of Entrée and Appetizer Selections by Age Group and Interest in Sustainability
eReferences

Supplement 3.
Data Sharing Statement
