# Supplementary material for: Ecolabels and the Healthfulness and Carbon Footprint of Restaurant Meal Selections: A Randomized Clinical Trial
Source: JAMA Netw Open. 2025 Aug 4;8(8):e2524773. doi: 10.1001/jamanetworkopen.2025.24773 (PMC12322791; doi:10.1001/jamanetworkopen.2025.24773)
Supplement: Supplement 1. — Trial Protocol [file jamanetwopen-e2524773-s001.pdf]

Effect of ecolabels on the healthfulness and carbon footprint of restaurant selections: A randomized clinical trial

Notes

Per journal instructions, this document contains the trial protocol and analysis plan as it was submitted to the Harvard Pilgrim Health Care Institute Institutional Review Board. We additionally pre-registered the analysis plan through ClinicalTrials.gov (NCT#06584539). Deviations from this plan are detailed in the main text. Note that this document includes protocol information for additional studies under the same IRB protocol as the randomized trial reported in the main text. We have also revised the original document to remove a copyright-protected image.

Foods Go Green: Prevalence and Influence of Environmental Sustainability Labels for Restaurant Foods
IRB Protocol

Background and Significance

Americans are increasingly concerned about the environmental impacts of their food choices, with more than 3 in 4 Americans identifying environmental sustainability as an important factor driving their decisions about what foods to buy.[1] Interest in promoting environmental sustainability is even higher among certain groups, such as younger adults.[2–6] As Americans' focus on sustainable food choices grows, retailers may seek to capitalize on this interest by marketing their products as more environmentally sustainable. One common marketing strategy is labeling products with "eco-labels" that signal when a product is more environmentally sustainable than other options. This strategy has become increasingly popular in restaurants. For example, several large chain restaurants have adopted eco-labels on their menus (e.g. "earth friendly pick" at sweetgreen) that identify more sustainable foods.[7–11]

Because more environmentally sustainable foods tend to be healthier than less sustainable foods,[12,13] eco-labels could have the additional benefit of nudging environmentally-conscious consumers toward healthier meal options in restaurants. This is a critical public health goal because restaurant foods, which contribute approximately one-third of Americans' daily calorie intake,[14] are typically higher in calories, saturated fat, and sodium; contain fewer fruits and vegetables; and are served in larger portions than foods prepared at home.[14,15] Eco-labels thus might shift consumers toward healthier restaurant choices – including consumers who are not motivated by health messaging. However, despite the growing popularity of eco-labels in restaurants, we know very little about their impact on diet.[16] For example, it is unknown whether the specific foods that display eco-labels are actually healthier options. Additionally, we have limited understanding of how eco-labels affect the healthfulness of food choices in restaurants, as almost no randomized trials of eco-labels have evaluated nutritional outcomes.[17] If eco-labels nudge consumers to select healthier foods, this would suggest new avenues for encouraging healthier dietary choices among a wider array of Americans. Alternatively, if eco-labels worsen the nutritional quality of consumers' food choices, this would suggest the need to modify label designs, for example by designing labels that communicate both sustainability and health effects of food.

The *overall objectives* of the proposed research are to 1) quantify the use of eco-labels on restaurant menus over time and evaluate the extent to which they are associated with nutritional quality and 2) evaluate how eco-labels affect the healthfulness of restaurant meal choices. Our *hypotheses* are that menu items with eco-labels will have higher nutritional quality than foods without eco-labels, and that eco-labels will be associated with healthier meal choices. We propose two aims:

Aim 1. Examine the prevalence of eco-labels on menu items (Aim 1A) and their association with the nutritional quality of foods (Aim 1B) in top-selling restaurants in the U.S.

Introduction. The first objective of this aim (1A) will examine the extent to which top-selling U.S. restaurants have placed eco-labels on menu items, as well as trends in use of these labels over time. The second objective (1B) will examine and compare the nutritional quality of restaurant items with an eco-label to similar items without an eco-label.

Sample. We will use data from Menustat, a database of restaurant items offered for sale at the top restaurants in the U.S. Every year since 2012, Menustat has identified the top ~100 restaurants by market share and compiled data on all items offered by these restaurants. The data include items' nutrition information (i.e., macro- and micronutrients), description, food group (e.g., entrees, desserts), and attributes describing how the item is displayed on menus ("display attributes," e.g., menu section, presence of labels). From 2012 to 2018, Menustat datasets were created and maintained by the New York City Department of Health and Mental Hygiene (NYC DOHMH) and posted publicly on menustat.org. Because of budget cuts and the COVID-19 pandemic, NYC DOHMH discontinued Menustat in 2019. To preserve Menustat as a publicly available data source, Jason Block's research team took over data collection and processing as part of an ongoing R01 on calorie labeling and is now operating the website. We will use Menustat datasets from 2012–2021 and restrict the sample to the 66 restaurants with data available every year; 2019–2021 data were collected by DPM staff and investigators. Data for these 66 restaurants are currently available for 2012–2020 and include n=182,825 item-year observations.

Eco-labels. For each restaurant menu item, we will identify whether the menu displayed an eco-label for that item by searching display attributes for terms associated with environmental sustainability (e.g., "meatless", "plant-based", "sustainable", "vegan"). We will refine and finalize the list of terms by conducting a literature review in partnership with a research librarian at the Harvard Countway Library.

Measures. Our primary outcomes will be the absolute and relative amounts of calories and nutrients of public health concern in menu items: sugar, saturated fat, sodium, and fiber. *Absolute* amounts of calories and nutrients reflect what customers actually buy while *relative* amounts of calories and nutrients reflect items' overall nutritional quality, which is strongly associated with health.[18] Relative amounts include calorie and nutrient densities (i.e., percent of calories from each nutrient; calorie and nutrients per 100g of food or beverage), as well as the percent of items that exceed internationally recognized thresholds for excessive levels of calories, sugar, saturated fat, and sodium.[19]

Analysis and power. *Prevalence of eco-labels (Aim 1A)*. We will determine the number of unique restaurant items offered each year that display an eco-label, in absolute terms and as a proportion of all foods offered. In a preliminary analysis among the 66 restaurants with continuously available data, we found that the number of items with "vegetarian" or "vegan" labels increased from 28 items (0.2% of all items) in 2012 to 253 (1.2%) in 2020 **(Figure 2)**.

Product healthfulness (Aim 1B). We will fit generalized linear models that regress the outcome of interest on eco-label status, adjusting for chain, year, and food group fixed effects. The coefficient for eco-label status will represent the average difference in each outcome for foods with an eco-label versus those without an eco-label within a restaurant chain, year, and food group. Models will use an identity link for continuous outcomes and a logistic link for binary outcomes (to calculate predicted probabilities). Based on previous Menustat studies, we anticipate sufficient power to detect small associations of eco-labels with nutritional quality. For example, an analysis of 2012–2018 Menustat data detected statistically significant differences in mean sugar content (g) between side dishes with (b=6.1g [95% CI: 5.3-6.9]) and without health-related ingredient claims (b=9.1g [95% CI: 7.3, 10.9], P-diff<0.001).[20] That analysis included only 301 items with the claim of interest, which is appreciably smaller than our sample (e.g., there are n=316 unique items with "vegan" or "vegetarian" attributes alone).

Figure 2. "Vegan" and "vegetarian" mentions in Menustat, 2012-2020

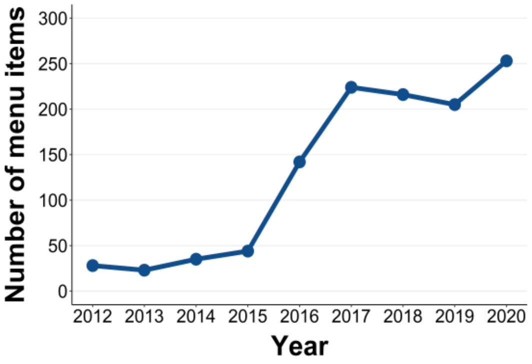

Aim 2a. Identify promising eco-label designs. Food retailers are increasingly using eco-labels to market the foods they sell. These labels could be a promising strategy for informing consumers about sustainable food options and reducing consumption of less-sustainable foods. However, debate remains about how to design eco-labels to maximize their ability to influence consumer behavior. For example, eco-labels adopted by restaurants such as the fast casual chain Just Salad communicate numeric information about the greenhouse gas emissions associated with producing each menu item, expressed as carbon dioxide (CO2) equivalents. By contrast, restaurants including Panera and Sweetgreen use positive (or "endorsement") logos to highlight menu items that have lower environmental impact, without providing numeric information. Another difference is whether labels feature text, icons, or both. We will examine how consumers respond to these different eco-label designs.

Aim 2b. New insights on optimal strategies for communicating about sugary drinks. Specifically, this study aims to examine consumer responses to traditional and counter-marketing messages discouraging sugary drink consumption, including effects on sugary drink consumption intentions and perceived weight stigma.

Participants: We will recruit a national convenience sample of ~2,150 US adults, with an oversample of young adults ages 18-29 to comprise 50% of total sample. Participants will be recruited by Cloud Research Prime Panels. Cloud Research is an online participant recruitment platform with access to over 100 million participants. Partnering with Cloud Research will allow us to easily reach the sample size we seek. Cloud Research carefully sources participants and uses vetting practices to prevent fraudulent respondents and ensure high data quality. Given the large sample size and diversity of participants in the Cloud Research panels, we anticipate that participants will comprise a variety of gender and minority identities.

Recruitment:

We will recruit participants through Cloud Research, a survey research firm we have worked with previously. Cloud Research integrates several online research panels common in market research. CloudResearch will recruit participants by emailing eligible panelists in their panels an invitation to participate in the survey. Panelists can opt-into the survey by following a hyperlink in

the email invitations. The link will take them to a Qualtrics survey programmed by our team containing this study's survey items. To avoid sample selection bias based on survey topic, CloudResearch uses only generic email subject lines such as "Survey Invitation." The content of the email message is likewise generic and includes only the study length and reward amount and type, as well as the opt-in link.

Consent:
The first page of the Qualtrics survey will display this study's electronic consent form. If interested in participating, participants will indicate their consent by clicking "I consent" and continuing with the survey.

Screening:
Participants must be age 18 or older to participate and must pass a "Captcha" screening question. The first two survey items after the consent form will query participants' age and ask them to complete the Captcha; participants who are ineligible will be re-directed to the survey termination page.

Survey:
The survey contains three main parts after the consent and screening questions: the experiment for Aim 2a, the experiment for Aim 2b, and standard demographic questions. In total, we anticipate the survey will take about 8-12 minutes to complete. Procedures for each part of the survey are described in more detail below.

First, participants will answer survey questions related to eco-labels. Participants will be randomized to 1 of 5 conditions:

1. Control condition: Participants will view neutral labels that do not mention sustainability.
2. Numeric text-only label condition: Participants will view designs describing the environmental impact of the food that contain text only,e.g., "1.4 KG CO2 per 100G".
3. Endorsement text-only label condition: Participants will view designs endorsing the low environmental impact of certain foods that contain text only, e.g. "Earth-Friendly".
4. Endorsement icon-only label condition: Participants will view designs endorsing the low environmental impact of certain foods that contain only icons, e.g., a picture of a small earth.
5. Endorsement text-plus-icon label condition: Participants will view designs endorsing the low environmental impact of certain foods that contain text and icons, e.g. "Earth-Friendly" alongside a picture of a small earth.

Participants will view 3 labels from their assigned condition. The stimuli are attached. For each label, we will first display the label by itself, then show the label in the context of a mock restaurant menu mimicking an excerpt of a Chili's menu. Then participants will answer questions about the label e.g., attention, emotional responses, believability; see attached survey. They will repeat this process for the second and third labels. Finally, to gain additional insight on optimal label design, we will ask participants to view and rate a variety of specific wording variations and specific icon designs.

In the second part of the survey, participants will be randomized to 1 of 3 arms:

1. Control: participants view a neutral message unrelated to sugary drinks about driving safety
2. Traditional health message: participants view a traditional health message about the health effects of sugary drinks
3. Counter-marketing message: participants view counter-marketing messages meant to expose deceptive marketing practices used by sugary drink companies.

Participants will view their randomly assigned message which will be mocked up as a series of Instagram posts and answer survey questions about the message, e.g., perceived effectiveness, emotional reactions, and their intentions to consume sugary drinks. The messages for each condition are shown below.

Finally, participants will answer standard demographic questions, e.g., gender, race/ethnicity, education. Participants will be debriefed as to the purpose of the study. After the survey is completed, participant will be routed to a "survey termination page" which will thank them for their time. They will be compensated by Cloud Research in an amount and form (e.g., cash, points redeemable for prizes) previously agreed upon between the participant and Cloud Research. Our study team will have no contact with research participants; Cloud Research will recruit and compensate the participant without our involvement. Additionally, our team will not collect or have access to any identifying information about participants. There will be no follow-up after the survey is complete.

Analysis
Aim 2a: Using a mixed model, we will regress outcomes on indicators for each label condition (i.e., the type of eco-label participants saw in the survey), excluding the control as the referent. We will treat the intercept as random to account for repeated measures within participants. Analyses will use the mixed models to estimate ADEs (differences in predicted means between groups) for each comparison of interest: (a) Each eco-label vs. control and (b) Each eco-label vs. one another. Given the exploratory nature of this study, we do not plan to adjust for multiple comparisons.

Aim 2b: We will regress outcomes on an indicator variable for message condition (i.e., the type of beverage message participants saw in the survey). Moderation analyses will add potential moderators and their interaction with message conditions to the primary model.

Aim 3. Effect of eco-labels on healthfulness of restaurant selections
Participants:
As with Aim 2, we will recruit a national sample of 3,100 US adults through Cloud Research Prime Panels, a survey research platform that provides access to millions of study participants and with whom we have worked previously.[21–25] Participants will be eligible if they reside in the US and are 18+ years old. Because younger adults may respond more strongly to eco-labels than middle-aged and older adults due to their greater interest in environmental sustainability,[2–6] we will require that approximately half of the sample (n=1,550) is comprised of young adults ages 18–29.

Recruitment:
We will recruit participants through Cloud Research, a survey research firm we have worked with previously. Cloud Research integrates several online research panels common in market research. CloudResearch will recruit participants by emailing eligible panelists in their panels an invitation to participate in the survey. Panelists can opt-into the survey by following a hyperlink in the email invitations. The link will take them to a Qualtrics survey programmed by our team containing this study's survey items. To avoid sample selection bias based on survey topic, CloudResearch uses only generic email subject lines such as "Survey Invitation." The content of the email message is likewise generic and includes only the study length and reward amount and type, as well as the opt-in link.

Consent:

The first page of the Qualtrics survey will display this study's electronic consent form. If interested in participating, participants will indicate their consent by clicking "I consent" and continuing with the survey.

Screening:
Participants must be age 18 or older to participate and must pass a "Captcha" screening question. The first two survey items after the consent form will query participants' age and ask them to complete the Captcha; participants who are ineligible will be re-directed to the survey termination page.

Procedures:
We will experimentally test the extent to which eco-labels affect the healthfulness of consumers' restaurant meal choices. Participants will choose a restaurant meal from Chili's. We selected Chili's because it is consistently among the top-selling restaurants in the US[26,27] and because it offers a wide range of foods, including both more and less environmentally sustainable options (e.g., salads, vegetarian entrees, steaks, burgers). Participants will view a menu adapted from Chili's online ordering webpage. They will be instructed to imagine they are ordering a meal for themselves and to select a meal they would like to eat. Participants will be told that 25 of them will be chosen at random to have their selection delivered to an address of their choosing. In reality, at the end of the study, these participants will instead receive a gift card of equivalent value. This very minor deception enables us to incentivize participants to choose foods they actually want to eat and to minimize social desirability bias. After selecting their preferred food from Chili's, participants will respond to survey questions programmed in Qualtrics (see *Measures*).

Randomization and trial arms. Participants will be randomly assigned to 1 of 2 trial arms: control or eco-labels. In the control arm, participants will view the existing Chili's menu with no additional labels added. For the eco-labels arm, we will adapt the Chili's menu to add eco-labels to foods with lower carbon footprint (i.e., that generate lower greenhouse gas emissions to produce; we chose to focus on greenhouse gas emissions because they are best-documented environmental impact of food[12]). We will approximate the carbon footprint for each menu item by linking items to their closest match in dataFRIENDS, a database of the carbon footprint associated with production of a given food. The database includes carbon footprint information for more than 10,000 specific food items consumed by participants in the National Health and Nutrition Examination Surveys, including from restaurants.[28,29] We will add eco-labels to foods that are below the 50th percentile of carbon footprint within their menu category (e.g., entrees, sides).[30–32] This approach to labeling mirrors how restaurants currently use eco-labels[7,8] and thus offers a realistic test of how these labels affect restaurant meal choices.

Stimuli. We will develop an eco-label for Aim 3 based on the results of Aim 2a. Specifically, the eco-label will include text and an icon. The text will read, "Environmentally friendly" and the icon will look like an earth. Aim 2a found this design was especially effective.

Measures. The primary outcome will be the healthfulness of the menu item the participant selected, assessed using the United Kingdom's OfCom Nutrient Profiling Model Score.[33,34] Prospective studies demonstrate that healthier diets as identified by this model are associated with lower risk of obesity[35] and cardiovascular disease.[36] Foods lower in calories, saturated fat, sugar, and sodium and higher in fiber, protein, and fruit, vegetable, and nut content are scored as healthier.[33,34] We will calculate healthfulness scores for each menu item using nutrition information from the restaurant's website.[37] We will measure other secondary outcomes related to carbon footprints, nutrients, and psychological outcomes.

Statistical analysis and power:

To evaluate the impact of eco-labels on healthfulness of restaurant choices, we will compare mean healthfulness scores for the two trial arms (eco-labels vs. control) using a two-sample *t*-test. Power analyses indicated that a sample size of 3,100 will provide 90% power to detect a standardized mean difference between groups of d=0.12. This estimate of effect size is conservative based on prior studies of environmental[32,38,39] and health[40–42] labels. We will also test for potential effect modification of eco-labels on healthfulness of restaurants choices by age (young adult [18-29] vs. middle and older adults [30 and older]).

Impact:
Aim 3 will indicate how eco-labels affect the healthfulness of consumers' food choices. This will illuminate the potential for eco-labels to improve population health.

Appendix

Stimuli For Aim 2a Study of Menu Labels

Notes: Participants will be assigned to 1 of the 5 label conditions below. They will view all 3 labels from their condition.

1. **Control condition**
   a.
   b. AVAILABLE
   c. SCAN HERE

2. **Numeric, text-only sustainability label**
   a. 1.4 KG CO_2 PER ITEM
   b. 1.4 KG CO_2 PER 100 G
   c. 1.4 KG CO_2 PER 100 CAL

3. **Interpretative, text-only sustainability label**
   a. LOW CLIMATE IMPACT
   b. EARTH-FRIENDLY
   c. SUSTAINABLE CHOICE

4. **Interpretative, icon-only sustainability label**
    a. 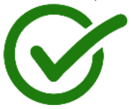
    b. 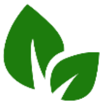
    c. 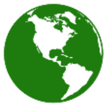
5. **Interpretative, text-plus-icon sustainability label**
    a. 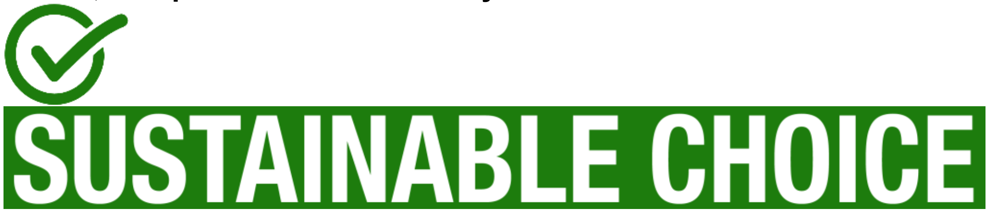

    b. 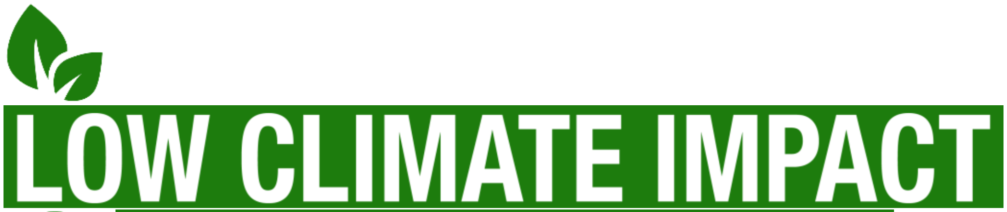

    c. 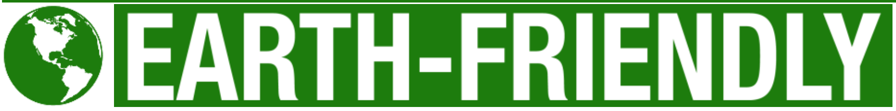

After participants rate the above labels from their condition, they will also rate several labels that vary the wording and icon used. Specifically, they will view 6 labels mimicking the labels shown in Condition 3 above (Interpretative, text-only sustainability label). These labels will use the following wording:

1. Low carbon
2. Sustainable choice
3. Earth-friendly
4. Environmentally-friendly
5. Climate-friendly
6. Low climate impact

Finally, participants will view 4 labels mimicking the labels shown in Condition 4 above (Interpretative, icon-only sustainability label). These labels will used the following icons:

1. 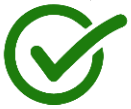
2. 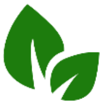

3. 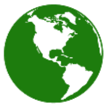
4.

Stimuli For Aim 2b Study of Beverage Messages

Messages

Notes: Participants will be assigned to 1 of the 3 message conditions below. They will view all 4 messages from their condition in the order shown below.

1. **Control Condition** [Note: Text adapted from the National Highway Traffic Safety Administration's Speed Prevention marketing materials[43]]. Messages:
    a. Driving over the speed limit can be costly. If law enforcement pulls you over for speeding, you could get a ticket with a fine. The cost of your insurance could also increase.
    b. The speed limit is the law. Speeding may seem like the faster option, but, ultimately, speeding slows you down.
    c. Speeding catches up with you. Speeding fatalities have continued to increase over the past few years. Even advances in car safety can't keep you safe from the dangers of driving too fast. Studies show that speeding is a contributing factor in nearly 1 in 3 fatal crashes in the US.
    d. Don't speed! Driving the speed limit is a simple way to prevent costly tickets and reduce risk of a crash.

2. **Traditional Health Message Condition** [Note: Text adapted from the 'Rethink Sugary Drink' campaign used in Australia,[44] the 'Choose Healthy Drinks Awareness Campaign' in San Mateo County, CA[45] the 'Rethink Your Drink' campaign developed by the California Department of Public Health,[46] the Center for Disease Control and Prevention's 'Rethink Your Drink' advertisements,[47] and New York City's sugary drink campaigns[48]]. Messages:
    a. Sodas, sports drinks, and fruit drinks are loaded with sugar. In fact, sugary drinks are the single largest source of added sugar in our diet.
    b. A single bottle of soda has 22 packs of sugar. You wouldn't eat 22 packs of sugar, so why would you drink them?
    c. Sugary drinks might look tempting. But dozens of studies show that sugary drinks are toxic to your health. Just one sugary drink per day can lead to 10 pounds of weight gain per year. Being overweight or obese puts you at greater risk of heart disease, type 2 diabetes, and some cancers.
    d. Don't pour on the pounds! Take action—it's as simple as drinking fewer sugary drinks.

3. **Counter-Marketing Message Condition** [Note: Text adapted from prior campaigns including San Francisco's 'Open Truth' campaign, Hawaii's 'Sweet Lies' campaign[49], and

New York City's 'Sour Side of Sweet' campaign,[50] as well as research on effective counter-marketing campaigns[51–54]]. Messages:

a. Sodas, sports drinks, and fruit drinks are loaded with sugar. That's on purpose – just like cigarettes are designed to be addictive, the beverage industry has engineered sugary drinks to make us crave them.
b. What's worse is that the beverage industry intentionally targets kids with kid-friendly marketing to get them hooked on sugary drinks for life.
c. Big brands like Gatorade, Coke, and Pepsi use athlete and celebrity endorsements to manipulate you into buying their products. What these companies don't want you to know is that sugary drinks are toxic to your health. Just *one* sugary drink per day can increase your risk of type 2 diabetes and heart damage.
d. Don't let these companies make a profit by making you sick. Take action—it's simple as drinking fewer sugary drinks.

Stimuli For Aim 3 Study of Eco-Labels

Notes: Participants will be assigned to 1 of the 2 label conditions below.

1. 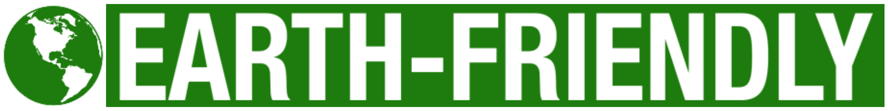

2. 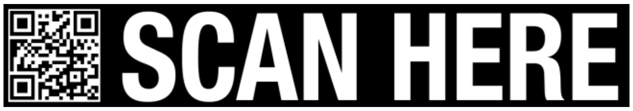

References

1. Cone Communications. 2014 Cone Communications Food Issues Trend Tracker – Cone. Published 2014. Accessed September 28, 2022. https://conecomm.com/2014-food-issues-trend-tracker/

2. First Insight. *The State of Consumer Spending: Gen Z Shoppers Demand Sustainability Retail*. First Insight, Inc; 2020. Accessed June 26, 2020. https://www.firstinsight.com/white-papers-posts/gen-z-shoppers-demand-sustainability

3. Hamel L, Lopes L, Muñana C, Brodie M. *The Kaiser Family Foundation/Washington Post Climate Change Survey*. Kaiser Family Foundation; 2019. Accessed June 26, 2020. https://www.kff.org/other/report/the-kaiser-family-foundation-washington-post-climate-change-survey/

4. Reinhart R. Global Warming Age Gap: Younger Americans Most Worried. Gallup News. Published May 11, 2018. Accessed June 30, 2020. https://news.gallup.com/poll/234314/global-warming-age-gap-younger-americans-worried.aspx

5. Saad L. Americans as Concerned as Ever About Global Warming. Gallup News. Published 2019. Accessed June 30, 2020. https://news.gallup.com/poll/248027/americans-concerned-ever-global-warming.aspx

6. Amnesty International. *Amnesty International "Future of Humanity" Survey*. Amnesty International; 2019. Accessed May 7, 2021. https://app.box.com/s/553divf5yaxizffhzqtu8ue0fsvnslj1/file/571721723499

7. sweetgreen. sweetgreen: Order Online. Published 2022. Accessed September 23, 2022. https://order.sweetgreen.com/

8. Lucas A. Panera Bread to label entrees as climate friendly. CNBC. Published October 4, 2020. Accessed September 23, 2022. https://www.cnbc.com/2020/10/14/panera-bread-to-label-entrees-as-climate-friendly.html

9. Morrison O. Carbon labels for food businesses and restaurants launched in 'world first.' foodnavigator.com. Published August 5, 2021. Accessed September 26, 2022. https://www.foodnavigator.com/Article/2021/08/05/Carbon-labels-for-food-businesses-and-restaurants-launched-in-world-first

10. George S. Wahaca adds carbon labels to restaurant menus - edie. https://www.edie.net/. Published April 7, 2022. Accessed September 23, 2022. https://www.edie.net/wahaca-adds-carbon-labels-to-restaurant-menus/

11. Collings R. Fast-Casual Restaurants Add Carbon Labeling to Menus in an Effort to Be More Sustainable. Published March 22, 2021. Accessed September 26, 2022. https://www.adweek.com/commerce/fast-casual-restaurants-add-carbon-labeling-to-menus-in-an-effort-to-be-more-sustainable/

12. Willett W, Rockström J, Loken B, et al. Food in the Anthropocene: the EAT–Lancet Commission on healthy diets from sustainable food systems. *The Lancet*. 2019;393(10170):447-492.

13. Clark MA, Springmann M, Hill J, Tilman D. Multiple health and environmental impacts of foods. *Proc Natl Acad Sci USA*. 2019;116(46):23357. doi:10.1073/pnas.1906908116

14. Saksena MJ, Okrent AM, Anekwe TD, et al. *America's Eating Habits: Food Away From Home*. United States Department of Agriculture, Economic Research Service; 2018. Accessed August 15, 2020. https://www.ers.usda.gov/publications/pub-details/?pubid=90227

15. Lin BH, Cuthrie J. *Nutritional Quality of Food Prepared at Home and Away From Home*. United States Department of Agriculture Economic Research Service; 2012. Accessed December 30, 2016. https://www.ers.usda.gov/publications/pub-details/?pubid=43699

16. Brown KA, Harris F, Potter C, Knai C. The future of environmental sustainability labelling on food products. *The Lancet Planetary Health*. 2020;4(4):e137-e138. doi:10.1016/S2542-5196(20)30074-7

17. Potter C, Bastounis A, Hartmann-Boyce J, et al. The effects of environmental sustainability labels on selection, purchase, and consumption of food and drink products: A systematic review. *Environment and behavior*. 2021;53(8):891-925.

18. Willet WC. Implications of Total Energy Intake for Epidemiologic Analyses. In: Willett WC, ed. *Nutritional Epidemiology*. 3rd ed. Oxford University Press; 2013:260-286.

19. Corvalán C, Reyes M, Garmendia ML, Uauy R. Structural responses to the obesity and non-communicable diseases epidemic: The Chilean Law of Food Labeling and Advertising. *Obes Rev*. 2013;14(Supple 2):79-87. doi:10.1111/obr.12099

20. Krobath DM, Masters WA, Mueller MP. Association between restaurant menu item descriptions and their nutrient content. *Am J Prev Med*. 2021;60(2):232-240. doi:10.1016/j.amepre.2020.08.027

21. Hall MG, Lazard AJ, Grummon AH, et al. Designing warnings for sugary drinks: A randomized experiment with Latino and non-Latino parents. *Prev Med*. 2021;148:106562. doi:10.1016/j.ypmed.2021.106562

22. Grummon AH, Ruggles PR, Greenfield TK, Hall MG. Designing effective alcohol warnings: Consumer reactions to icons and health topics. *Am J Prev Med*. Published online 2022. doi:https://doi.org/10.1016/j.amepre.2022.09.006

23. Grummon AH, Musicus AA, Moran AJ, Salvia MG, Rimm EB. Consumer reactions to positive and negative front-of-package food labels. *Am J Prev Med*. Published online 2022. doi:10.1016/j.amepre.2022.08.014

24. Grummon AH, Sokol R, Goodman D, et al. Storybooks about healthy beverage consumption: Effects in an online randomized experiment with parents. *Am J Prev Med*. 2022;62(2):183-192.

25. Grummon AH, Musicus AA, Salvia MG, Thorndike AN, Rimm EB. Impact of health, environmental, and animal welfare messages discouraging red meat consumption: An online randomized experiment. *J Acad Nutr Diet*. Published online October 9, 2022. doi:10.1016/j.jand.2022.10.007

26. FSR Magazine. The 50 Top-Grossing Full-Service Restaurants in America. FSR magazine. Published August 2020. Accessed September 22, 2022. https://www.fsrmagazine.com/chain-restaurants/50-top-grossing-full-service-restaurants-america

27. Duncan N. Full-Service Restaurant Chains, Ranked by Sales. FSR magazine. Published 08/22. Accessed September 22, 2022. https://www.fsrmagazine.com/2022-fsr-50/rankings

28. Heller MC, Willits-Smith A, Meyer R, Keoleian GA, Rose D. Greenhouse gas emissions and energy use associated with production of individual self-selected US diets. *Environmental Research Letters*. 2018;13(4):044004.

29. Rose D, Willits-Smith AM, Heller MC. Single-item substitutions can substantially reduce the carbon and water scarcity footprints of US diets. *The American Journal of Clinical Nutrition*. 2022;115(2):378-387. doi:10.1093/ajcn/nqab338

30. Leach AM, Emery KA, Gephart J, et al. Environmental impact food labels combining carbon, nitrogen, and water footprints. *Food Policy*. 2016;61:213-223.

31. Piester HE, DeRieux CM, Tucker J, Buttrick NR, Galloway JN, Wilson TD. "I'll try the veggie burger": Increasing purchases of sustainable foods with information about sustainability and taste. *Appetite*. 2020;155:104842.

32. Osman M, Thornton K. Traffic light labelling of meals to promote sustainable consumption and healthy eating. *Appetite*. 2019;138:60-71. doi:10.1016/j.appet.2019.03.015

33. Rayner M. Nutrient profiling for regulatory purposes. *Proceedings of the Nutrition Society*. 2017;76(3):230-236. doi:10.1017/S0029665117000362

34. Rayner M, Scarborough P, Lobstein T. *The UK Ofcom Nutrient Profiling Model: Defining "healthy" and "Unhealthy" Foods and Drinks for TV Advertising to Children*.; 2009. Accessed June 12, 2022. https://docs.google.com/viewer?url=https%3A%2F%2Fwww.ndph.ox.ac.uk%2Fcpnp%2Ffiles%2Fabout%2Fuk-ofcom-nutrient-profile-model.pdf

35. Egnell M, Seconda L, Neal B, et al. Prospective associations of the original Food Standards Agency nutrient profiling system and three variants with weight gain, overweight and obesity risk: results from the French NutriNet-Santé cohort. *Br J Nutr*. 2021;125(8):902-914. doi:10.1017/S0007114520003384

36. Adriouch S, Julia C, Kesse-Guyot E, et al. Prospective association between a dietary quality index based on a nutrient profiling system and cardiovascular disease risk. *Eur J Prev Cardiol*. 2016;23(15):1669-1676. doi:10.1177/2047487316640659

37. Chili's. Nutrition Info - Chili's. Published 2022. Accessed September 28, 2022. https://www.chilis.com/media/docs/chilis-nutrition-menu-generic

38. Hallstein E, Villas-Boas SB. Can household consumers save the wild fish? Lessons from a sustainable seafood advisory. *Journal of Environmental Economics and Management*. 2013;66(1):52-71.

39. Jaffry S, Pickering H, Ghulam Y, Whitmarsh D, Wattage P. Consumer choices for quality and sustainability labelled seafood products in the UK. *Food Policy*. 2004;29(3):215-228.

40. Kaur A, Scarborough P, Rayner M. A systematic review, and meta-analyses, of the impact of health-related claims on dietary choices. *International Journal of Behavioral Nutrition and Physical Activity*. 2017;14(1):93. doi:10.1186/s12966-017-0548-1

41. Hall MG, Lazard AJ, Grummon AH, Mendel JR, Taillie LS. The impact of front-of-package claims, fruit images, and health warnings on consumers' perceptions of sugar-sweetened fruit drinks: Three randomized experiments. *Prev Med*. 2020;132:105998. doi:10.1016/j.ypmed.2020.105998

42. Hall MG, Lazard AJ, Higgins IC, et al. Nutrition-related claims lead parents to choose less healthy drinks for young children: a randomized trial in a virtual convenience store. *The American Journal of Clinical Nutrition*. 2022;115(4):1144-1154.

43. US Department of Transportation National Highway Traffic Safety Administration. Speeding Wrecks Lives | Traffic Safety Marketing. Published 2023. Accessed April 16, 2023. https://www.trafficsafetymarketing.gov/get-materials/speed-prevention/speeding-wrecks-lives

44. Cancer Council Victoria. Sugary drink free tips & resources. Rethink Sugary Drink. Published 2023. Accessed March 7, 2023. https://www.rethinksugarydrink.org.au/tips-resources

45. Get Healthy San Mateo County. Choose Healthy Drinks Awareness Campaign. Get Healthy San Mateo County. Published March 22, 2016. Accessed March 7, 2023. https://www.gethealthysmc.org/general-information/choose-healthy-drinks-awareness-campaign

46. California Department of Public Health. Make Every Sip Count. Published 2019. Accessed March 7, 2023. https://docs.google.com/viewer?url=https%3A%2F%2Fwww.cdph.ca.gov%2FPrograms%2FCCDPHP%2FDCDIC%2FNEOPB%2FCDPH%2520Document%2520Library%2FRYDmakeeverysipcount.pdf

47. Centers for Disease Control and Prevention. Rethink Your Drink Award Submission. Centers for Disease Control and Prevention. Published April 4, 2022. Accessed March 7, 2023. https://www.cdc.gov/nccdphp/dnpao/multimedia/rethink-your-drink/index.html

48. Kansagra SM, Kennelly MO, Nonas CA, et al. Reducing sugary drink consumption: New York City's approach. *Am J Public Health*. 2015;105(4):e61-e64.

49. Hawaii State Department of Health. Sweet Lies. Living Healthy Hawaii. Published 2022. Accessed March 7, 2023. https://livinghealthy.hawaii.gov/sweetlies/

50. Clapp J. NYC Sugary Drink Campaigns. Presented at: August 21, 2019. Accessed March 7, 2023. https://docs.google.com/viewer?url=https%3A%2F%2Fwww.sfdph.org%2Fdph%2Ffiles%2FSDDTAC%2FNYC%2520SSB%2520media%2520082019.pdf

51. Bryan CJ, Yeager DS, Hinojosa CP, et al. Harnessing adolescent values to motivate healthier eating. *Proceedings of the National Academy of Sciences*. 2016;113(39):10830-10835. doi:10.1073/pnas.1604586113

52. Bryan CJ, Yeager DS, Hinojosa CP. A values-alignment intervention protects adolescents from the effects of food marketing. *Nature human behaviour*. 2019;3(6):596-603. doi:10.1038/s41562-019-0586-6

53. Palmedo PC, Dorfman L, Garza S, Murphy E, Freudenberg N. Countermarketing alcohol and unhealthy food: an effective strategy for preventing noncommunicable diseases? Lessons from tobacco. *Annual Review of Public Health*. 2017;38:119-144.

54. Palmedo PC, Flores S, Castillo K, Byrne-Zaaloff M, Moltzen K. Exploring Countermarketing Messages to Reduce Youth Sugar-Sweetened Beverage Consumption in The Bronx, NY. *Social Marketing Quarterly*. 2022;28(4):274-287.
