# Supplementary material for: Ecolabels and the Healthfulness and Carbon Footprint of Restaurant Meal Selections: A Randomized Clinical Trial
Source: JAMA Netw Open. 2025 Aug 4;8(8):e2524773. doi: 10.1001/jamanetworkopen.2025.24773 (PMC12322791; doi:10.1001/jamanetworkopen.2025.24773)
Supplement: Supplement 2. — eMethods. eFigure. Acceptability of Ecolabels, n = 3,147 US Adults eTable 1. Characteristics of Menu Items Shown in the Experiment eTable 2. Survey Measures Used in Experiment eTable 3. Comparison of Characteristics of the Study Sample (n = 3,147 US Adults) to National Estimates eTable 4. Impact of Ecolabels on Healthfulness of Entrée and Appetizer Selections by Age Group and Interest in Sustainability eReferences [file jamanetwopen-e2524773-s002.pdf]

# Supplemental Online Content

Grummon AH, Zeitlin AB, Lee CJY, et al. Ecolabels and the healthfulness and carbon footprint of restaurant meal selections: a randomized clinical trial. *JAMA Netw Open*. 2025;8(8):e2524773. doi:10.1001/jamanetworkopen.2025.24773

**eMethods.**

**eFigure.** Acceptability of Ecolabels, *n*=3,147 US Adults

**eTable 1.** Characteristics of Menu Items Shown in the Experiment

**eTable 2.** Survey Measures Used in Experiment

**eTable 3.** Comparison of Characteristics of the Study Sample (*n*=3,147 US Adults) to National Estimates

**eTable 4.** Impact of Ecolabels on Healthfulness of Entrée and Appetizer Selections by Age Group and Interest in Sustainability

**eReferences**

This supplemental material has been provided by the authors to give readers additional information about their work.

© 2025 Grummon AH et al. *JAMA Network Open*

## eMethods

**Estimating carbon footprints.** We estimated carbon footprints of menu items using the Database of Food Recall Impacts on the Environment for Nutrition and Dietary Studies (dataFRIENDS),[1,2] which has been widely used in research studies.[2–5] First, we disaggregated each item (as needed) into food components and ingredients based on the item description and nutrition facts documentation published by the restaurant. We disaggregated in one of two ways, depending on the item. For some items, we used nutritional information the restaurant published about the individual components that make up multi-ingredient items. For example, the restaurant published nutrition information for the components of the chicken fajitas, including the calorie content of the individual components (e.g., grilled chicken, Mexican rice, flour tortillas, sour cream, etc.). For items that lacked this composition information, we used information on comparable items from other restaurant chains or made assumptions about ingredients based on the menu item description on the restaurant website. For example, the skillet chocolate chip cookie is listed as being served with vanilla ice cream and hot fudge, so we assumed this menu item included a chocolate chip cookie, vanilla ice cream, and hot fudge, even though these ingredients are not disaggregated in the restaurant's nutritional database. We then assigned weights to each ingredient such that their total calorie content equaled the calories reported for the item by the restaurant.

Next, we assigned USDA Food and Nutrient Database for Dietary Studies (FNDDS) food codes to each food component. FNDSS is a database that provides nutrient values for foods and beverages consumed in the US. It contains information regarding approximately 15,000 unique foods and beverages (each assigned a unique food code), allowing us to match the specific components and ingredients in the menu items in our study to the corresponding food codes in FNDSS. For example, we assigned the food code 24123301 for "chicken breast, grilled without sauce, skin not eaten" to the grilled chicken component of the chicken fajitas menu item. Likewise, we assigned the food code 58163405 for "Spanish rice, from restaurant" to the Mexican rice in the chicken fajitas, and so on for the remaining ingredients. This involved selecting the FNDSS food code that most closely matched the component or ingredient of interest, a process that may have introduced noise to our estimation of carbon footprints.

Next, we estimated the weight in grams of each ingredient or component. For nearly all food components, data on calorie content was available in FNDSS, but information on weight was not. For these components, we converted calorie content into weight in grams by using the FNDDS reference database,[6] which provides caloric data

per 100g of specific types of foods. We converted calorie content to weight using the following formula: restaurant's food component calories * (100 g / FNDDS reference food calories).

Next, we linked each food component (using FNDSS food codes) to greenhouse gas (GHG) emissions data, expressed in carbon dioxide equivalents ($CO_2$-eq) per 100g using dataFRIENDS,[1,2] which includes information on GHG emissions at the food-code-level. Finally, we multiplied GHG emissions of each food component in $CO_2$-eq per 100g with their corresponding weight in grams to yield the GHG emissions associated with producing the food component. To calculate the total GHG emissions per item, we summed the GHG emissions of the food components and ingredients associated with each menu item.

**Designing the restaurant menu.** We developed the restaurant menu displayed in this study based on a popular sit-down restaurant chain in the US. The menu displayed 37 items, including four appetizers, 22 entrees, three desserts, and eight beverages. The menu displayed up to four items per row. Items were shown underneath the headings displayed in **eTable 1**. The menu displayed each item with a photo, a short description (e.g., "Cajun Shrimp Pasta"), and calorie information, but no prices. By excluding price, we aimed to keep the focus on the items participants most wanted to order, regardless of the price. Because the menus displayed in both the ecolabels arm and the control arm did not display price, we do not anticipate the absence of price influenced the internal validity of the study.

© 2025 Grummon AH et al. *JAMA Network Open*

**eFigure.** Acceptability of ecolabels, *n*=3,128 US adults

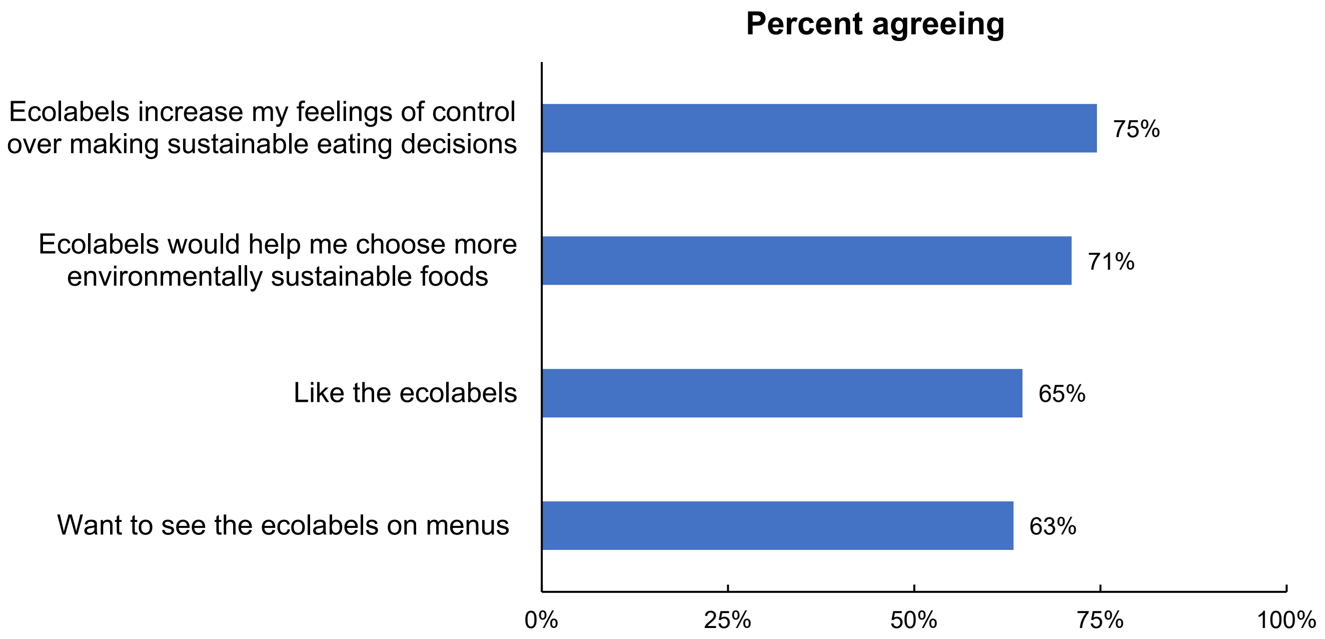


*Note*. Sample size is smaller than main sample due to missing data on these outcomes.

© 2025 Grummon AH et al. *JAMA Network Open*

**eTable 1.** Characteristics of menu items shown in the experiment

| Product | Greenhouse gas emissions per item, kgCO2 | Healthfulness, 0-100[a] | Eligible for ecolabel[b] |
|---|---|---|---|
| **Appetizers** | | | |
| Buffalo Chicken Wings | 1.64 | 40 | No |
| Chips, Guacamole & Salsa | 0.34 | 58 | Yes |
| Fried Mozzarella | 1.94 | 34 | No |
| Texas Cheese Fries | 0.98 | 42 | Yes |
| **Burgers & Sandwiches** | | | |
| Bacon Rancher Burger | 16.41 | 46 | No |
| Cheeseburger | 7.82 | 66 | No |
| Black Bean Cheeseburger | 0.77 | 70 | Yes |
| Crispy Chicken Sandwich | 0.80 | 52 | Yes |
| **Fajitas and Quesadillas** | | | |
| Steak Fajitas | 10.31 | 68 | No |
| Chicken Fajitas | 2.02 | 68 | No |
| Black Bean Veggie Fajitas | 1.40 | 68 | Yes |
| Brisket Quesadillas | 2.41 | 40 | No |
| **Grill & BBQ** | | | |
| Ancho Salmon | 0.88 | 70 | Yes |
| Ribeye Steak | 11.74 | 64 | No |
| Sirloin Steak | 9.56 | 70 | No |
| Full Order BBQ Ribs | 2.51 | 46 | No |
| **Pasta & Bowls** | | | |
| Cajun Shrimp Pasta | 7.57 | 50 | No |
| Cajun Chicken Pasta | 1.61 | 50 | Yes |
| Chipotle Shrimp Bowl | 5.35 | 66 | No |
| Chipotle Chicken Bowl | 1.12 | 68 | Yes |
| **Salads & Soups** | | | |
| Chicken Caesar Salad | 1.07 | 78 | Yes |
| Caesar Salad | 0.66 | 76 | Yes |
| Ancho Salmon Salad | 0.83 | 74 | Yes |
| House Salad | 0.29 | 74 | Yes |
| Original Chili | 3.25 | 48 | No |
| Loaded Baked Potato Soup | 0.87 | 56 | Yes |
| **Desserts** | | | |
| Skillet Chocolate Chip Cookie | 0.94 | 30 | - |
| Molten Chocolate Cake | 1.20 | 32 | - |
| Cheesecake | 0.85 | 32 | - |
| **Beverages** | | | |
| Coke | 0.03 | 66 | - |
| Diet Coke | 0.03 | 70 | - |
| Coke Zero | 0.03 | 70 | - |
| Sprite | 0.03 | 68 | - |
| Dr. Pepper | 0.03 | 66 | - |
| Minute Maid Lemonade | 0.08 | 66 | - |
| Unsweetened Tea | 0.04 | 70 | - |
| Mango Iced Tea | 0.03 | 70 | - |

[a]Healthfulness was assessed with the UK Ofcom Nutrient Profiling Model, converted to a 0-100 scale, where higher scores are healthier.[7,8]
[b]Only entrees and appetizers were eligible for ecolabels.

© 2025 Grummon AH et al. *JAMA Network Open*

**eTable 2.** Survey measures used in the trial

| Construct | Item [programming notes] | Response scale [programming notes] | Reference |
|---|---|---|---|
| | **Introduction (after consent)** | | |
| Captcha | Please complete the captcha.<br><br>[Display Captcha button] | | |
| Age | How old are you? | [Free response, #, restricted whole numbers to 0-115] | |
| | **Introduction to Eco-Labels Experiment** | | |
| Prompt | In this survey, you will see a restaurant menu. We will ask you to select items from this menu.<br><br>You will not be asked to spend your own money. However, you should make your selections carefully because we will randomly choose 25 respondents who will have their selections delivered to them. For those 25 respondents, this will be a “bonus” for completing this part of the survey. This means you should choose the menu items you most want because your selections could be delivered to you.<br><br>We care about the quality of our data. For us to get the most accurate information, it is important that you understand these instructions.<br><br>Please select the answer option that is correct based on the information you have read above: | [randomize order of responses]<br><br>1=I have to pay for the items I select with my own money<br>2=There is a chance I will be chosen to receive the items I selected<br>3=No participants in this study will receive the items they selected<br><br>[Participants cannot continue until they select response option 2. Custom validation text says, “Your response was incorrect. Please read the instructions carefully and try again.”] | |
| | **Menu** | | |
| Prompt | On the page below, you will see a restaurant menu. Imagine you are ordering from this restaurant for yourself. Take a few minutes to look at the menu. Then, click on the item or items that you would like to order. Please select at least 1 entrée or appetizer. After you have made your choices, move to the next page. Please make sure to scroll down to see all the menu items before you choose.<br><br>Select the item or items you want to order.<br><br>[Participants are randomized to one of two conditions: control or eco-label] | | |
| Prompt | [Display menu for assigned condition] | | |

© 2025 Grummon AH et al. *JAMA Network Open*

| Construct | Item<br>[programming notes] | Response scale<br>[programming notes] | Reference |
|---|---|---|---|
| | **Menu Selection Follow-Ups** | | |
| Noticing calorie labels | When you were selecting items to order, did you notice any calorie labels next to menu items? | 1=Yes<br>0=No | |
| Noticing trial labels | When you were selecting items to order, did you notice any other labels next to menu items? | 1=Yes<br>0=No | |
| Prompt | We are interested in how you selected foods from the menu we just showed you. When you chose what to order from the menu, how much did you think about each of the following characteristics?<br><br>[Format items below as a matrix; randomize order of attributes] | 1=Not at all<br>2=Very little<br>3=Somewhat<br>4=Quite a bit<br>5=A great deal | Adapted from Grummon et al. 2022[9] |
| Thinking about healthfulness | Healthfulness | | |
| Thinking about taste | Taste | | |
| Thinking about environmental sustainability | Environmental sustainability | | |
| | **Menu Item Perceptions – Sustainable Item** | | |
| Prompt | The next questions are about different menu items.<br><br>[Participants will view 1of the 13 sustainable items from their assigned condition] | | |
| Perceived sustainability – sustainable item | How environmentally sustainable do you think this item is?<br><br>[Display image of sustainable menu item with label from participant's condition] | 1=Not at all<br>2=A little<br>3=Somewhat<br>4=Very<br>5=Extremely | Adapted from Bollard 2016[10] |
| Perceived healthfulness – sustainable item | How healthy do you think this item is?<br><br>[Display image of sustainable menu item with label from participant's condition] | 1=Not at all<br>2=A little<br>3=Somewhat<br>4=Very<br>5=Extremely | Adapted from Bollard 2016[10] |
| Perceived tastiness – sustainable item | How tasty do you think this item is?<br><br>[Display image of sustainable menu item with label from participant's condition] | 1=Not at all<br>2=A little<br>3=Somewhat<br>4=Very<br>5=Extremely | Adapted from Bollard 2016[10] |
| | **Menu Item Perceptions – Unsustainable Item** | | |

© 2025 Grummon AH et al. *JAMA Network Open*

| Construct | Item<br>[programming notes] | Response scale<br>[programming notes] | Reference |
|---|---|---|---|
| Prompt | The next questions are about different menu items.<br><br>[Participants will view 1of the 13 unsustainable items from their assigned condition] | | |
| Perceived sustainability – unsustainable item | How environmentally sustainable do you think this item is?<br><br>[Display image of unsustainable menu item with label from participant's condition] | 1=Not at all<br>2=A little<br>3=Somewhat<br>4=Very<br>5=Extremely | Adapted from Bollard 2016[10] |
| Perceived healthfulness – unsustainable item | How healthy do you think this item is?<br><br>[Display image of unsustainable menu item with label from participant's condition] | 1=Not at all<br>2=A little<br>3=Somewhat<br>4=Very<br>5=Extremely | Adapted from Bollard 2016[10] |
| Perceived tastiness – unsustainable item | How tasty do you think this item is?<br><br>[Display image of unsustainable menu item with label from participant's condition] | 1=Not at all<br>2=A little<br>3=Somewhat<br>4=Very<br>5=Extremely | Adapted from Bollard 2016[10] |
| | **Eco-Label Acceptability** | | |
| Prompt | Some restaurants display labels next to menu items that are more environmentally sustainable, like the label below. You might have seen this label on the menu you saw earlier in the survey. The next questions are about this type of label.<br><br>[Display image of the eco-label by itself] | | |
| Prompt | Say how much you agree or disagree with the following statements. | | |
| Acceptability – Like ecolabel | I like this label. | 1=Strongly disagree<br>2=Somewhat disagree<br>3=Neither disagree nor agree<br>4=Somewhat agree<br>5=Strongly agree | Adapted from Vargas-Meza 2019[11] |
| Acceptability – Want to see ecolabel on menus | I want to see this label on menus next to foods that are more environmentally sustainable. | 1=Strongly disagree<br>2=Somewhat disagree<br>3=Neither disagree nor agree<br>4=Somewhat agree<br>5=Strongly agree | Adapted from Vargas-Meza 2019[11] |
| Acceptability – Ecolabel helps choose sustainable foods | This label would help me choose more environmentally sustainable foods. | 1=Strongly disagree<br>2=Somewhat disagree<br>3=Neither disagree nor agree<br>4=Somewhat agree<br>5=Strongly agree | Adapted from Vargas-Meza 2019[11] |

© 2025 Grummon AH et al. *JAMA Network Open*

| Construct | Item<br>[programming notes] | Response scale<br>[programming notes] | Reference |
|---|---|---|---|
| Acceptability – Ecolabel makes feel more in control | This label makes me feel more in control of making sustainable eating decisions. | 1=Strongly disagree<br>2=Somewhat disagree<br>3=Neither disagree nor agree<br>4=Somewhat agree<br>5=Strongly agree | Adapted from Acton 2018[12] |
| | **Demographics** | | |
| Prompt | **We are asking the questions in the next section to better understand who completed our survey.** | | |
| Gender | How do you identify? | 1=Woman<br>2=Man<br>3=Non-binary<br>4=Prefer to self-describe: ______ | New |
| Combined race and ethnicity | What is your race or ethnicity? (check all that apply) | [check all that apply]<br>1= American Indian or Alaska Native<br>2=Asian<br>3=Black or African American<br>4=Hispanic, Latino, or Spanish origin<br>5=Middle Eastern or North African<br>6=Native Hawaiian or other Pacific Islander<br>7=White<br>8=Some other race or ethnicity (please specify): _____ | Modified proposed U.S. Census item[12] |
| Education | What is the highest degree or level of school you have completed? | 1=Less than high school<br>2=High school graduate (or GED)<br>3=Some college or technical school<br>4=Associate's degree<br>5=Bachelor's degree<br>6=Graduate or professional degree | |
| Prompt | **Say how much you agree or disagree with the statements below.** | | |

© 2025 Grummon AH et al. *JAMA Network Open*

| Construct | Item [programming notes] | Response scale [programming notes] | Reference |
|---|---|---|---|
| GREEN scale | It is important to me that the products I use do not harm the environment. | 1 = Strongly disagree<br>2 = Somewhat disagree<br>3 = Neither agree nor disagree<br>4 = Somewhat agree<br>5 = Strongly agree | Haws et al., 2014[13] |
| GREEN scale | I consider the potential environmental impact of my actions when making many of my decisions. | 1 = Strongly disagree<br>2 = Somewhat disagree<br>3 = Neither agree nor disagree<br>4 = Somewhat agree<br>5 = Strongly agree | Haws et al., 2014[13] |
| GREEN scale | My purchase habits are affected by my concern for the environment. | 1 = Strongly disagree<br>2 = Somewhat disagree<br>3 = Neither agree nor disagree<br>4 = Somewhat agree<br>5 = Strongly agree | Haws et al., 2014[13] |
| GREEN scale | I am concerned about wasting the resources of our planet. | 1 = Strongly disagree<br>2 = Somewhat disagree<br>3 = Neither agree nor disagree<br>4 = Somewhat agree<br>5 = Strongly agree | Haws et al., 2014[13] |
| GREEN scale | I would describe myself as environmentally responsible. | 1 = Strongly disagree<br>2 = Somewhat disagree<br>3 = Neither agree nor disagree<br>4 = Somewhat agree<br>5 = Strongly agree | Haws et al., 2014[13] |
| GREEN scale | I am willing to be inconvenienced in order to take actions that are more environmentally friendly. | 1 = Strongly disagree<br>2 = Somewhat disagree<br>3 = Neither agree nor disagree<br>4 = Somewhat agree<br>5 = Strongly agree | Haws et al., 2014[13] |
| Party identification | Do you consider yourself a Democrat, a Republican, an Independent, or something else? | 1=Strong Democrat<br>2=Moderate Democrat<br>3=Lean Democrat<br>4=Lean Republican<br>5=Moderate Republican<br>6=Strong Republican<br>7=Independent<br>8=Something else: ____ | NORC AmeriSpeak TESS, adapted |
| Household size | How many people are in your household, including you? | [# of people [restricted to 1-20, whole numbers] | |

© 2025 Grummon AH et al. *JAMA Network Open*

| Construct | Item<br>[programming notes] | Response scale<br>[programming notes] | Reference |
|---|---|---|---|
| Children | How many children (ages 0-18) currently live in your household? | ____ [#, restricted to 0-15, whole numbers] | |
| Income | Which of the following categories best describes your total household income before taxes in the last 12 months? | 1=Less than $10,000<br>2=$10,000 to $14,999<br>3=$15,000 to $24,999<br>4=$25,000 to $34,999<br>5=$35,000 to $49,999<br>6=$50,000 to $74,999<br>7=$75,000 to $99,999<br>8=$100,000 to $149,999<br>9=$150,000 to $199,999<br>10=$200,000 or more | |

© 2025 Grummon AH et al. *JAMA Network Open*

**eTable 3.** Comparison of characteristics of the study sample (*n*=3,147 US adults) to national estimates

| | Study sample % | National estimate % |
|---|---|---|
| Age | | |
| 18-29 years | 50% | 20% |
| 30-44 years | 30% | 26% |
| 45-59 years | 14% | 23% |
| 60 years or older | 6% | 31% |
| Gender | | |
| Woman | 50% | 51% |
| Man | 50% | 49% |
| Non-binary or another gender | 0.4% | NA |
| Race and ethnicity | | |
| American Indian or Alaska Native, not Hispanic or Latino | 0.4% | 1% |
| Asian, Native Hawaiian, or Pacific Islander, not Hispanic or Latino | 9% | 6% |
| Black or African American, not Hispanic or Latino | 14% | 12% |
| Hispanic, Latino, or Spanish origin | 9% | 18% |
| Middle Eastern or North African, not Hispanic or Latino | 0.3% | NA |
| White, Not Hispanic or Latino | 63% | 60% |
| Another race or multi-racial, not Hispanic or Latino | 3% | 4% |
| Education | | |
| High school diploma or less | 14% | 37% |
| Some college | 19% | 20% |
| College graduate or associates degree | 52% | 29% |
| Graduate degree | 14% | 13% |
| Household income, annual | | |
| $0 to $24,999 | 11% | 14% |
| $25,000 to $49,999 | 21% | 17% |
| $50,000 to $74,999 | 22% | 16% |
| $75,000 or more | 46% | 53% |

[a]National estimates for age, gender, race and ethnicity, and education are survey-weighted estimates among adults (ages 18-years and older) in the 2023 American Community Survey (ACS) 1-year Public Use Microdata Sample (PUMS).[14] National estimate of % of people identifying as non-binary or another gender is listed as “NA” because the 2023 ACS did not include an option for people to identify as non-binary or another gender. National estimate of % of people identifying as Middle Eastern or North African is listed as “NA” because the 2023 ACS did not provide data on the proportion of respondents identifying in this category. National estimates for income are from the Current Population Survey, 2023.[15]

© 2025 Grummon AH et al. *JAMA Network Open*

**eTable 4.** Impact of ecolabels on healthfulness of entrée and appetizer selections by age group and interest in sustainability

| Characteristic | Difference, eco-label vs. control ADE | (95% CI) | *p* for interaction[a] |
|---|---|---|---|
| Age | | | |
| 18-29 | 0.59 | (-0.30, 1.47) | .66 |
| 30+ | 0.31 | (-0.57, 1.19) | |
| Interest in sustainability,[b] mean=3.42 (SD=0.93) | | | |
| Mean – 1 SD | 0.11 | (-0.78, 1.01) | .54 |
| Mean | 0.31 | (-0.33, 0.94) | |
| Mean + 1 SD | 0.51 | (-0.39, 1.41) | |

*Abbreviations.* ADE, average differential effect.
[a]*p* for interaction is for Wald tests of the joint significance of the coefficients on all interaction terms.
[b]Measured with the GREEN scale.[13]

© 2025 Grummon AH et al. *JAMA Network Open*

**eReferences**


1. University of Michigan Center for Sustainable Systems. Database of Food Impacts on the Environment for Linking to Diets (dataFIELD). 2017. Accessed August 3, 2020. http://css.umich.edu/page/datafield

2. Grummon AH, Lee CJY, Robinson TN, Rimm EB, Rose D. Simple dietary substitutions can reduce carbon footprints and improve dietary quality across diverse segments of the US population. *Nat Food*. Published online October 26, 2023:1-12. doi:10.1038/s43016-023-00864-0

3. Rose D, Willits-Smith AM, Heller MC. Single-item substitutions can substantially reduce the carbon and water scarcity footprints of US diets. *The American Journal of Clinical Nutrition*. 2022;115(2):378-387.

4. Rose D, Heller MC, Willits-Smith AM, Meyer RJ. Carbon footprint of self-selected US diets: nutritional, demographic, and behavioral correlates. *The American journal of clinical nutrition*. 2019;109(3):526-534.

5. Willits-Smith A, Aranda R, Heller MC, Rose D. Addressing the carbon footprint, healthfulness, and costs of self-selected diets in the USA: a population-based cross-sectional study. *The Lancet Planetary Health*. 2020;4(3):e98-e106. doi:10.1016/S2542-5196(20)30055-3

6. United States Department of Agriculture, Agricultural Research Service. FNDDS Documentation and Databases. October 7, 2024. Accessed December 16, 2024. https://www.ars.usda.gov/northeast-area/beltsville-md-bhnrc/beltsville-human-nutrition-research-center/food-surveys-research-group/docs/fndds-download-databases/

7. Rayner M, Scarborough P, Lobstein T. *The UK Ofcom Nutrient Profiling Model: Defining "healthy" and "Unhealthy" Foods and Drinks for TV Advertising to Children*.; 2009. Accessed June 12, 2022. https://docs.google.com/viewer?url=https%3A%2F%2Fwww.ndph.ox.ac.uk%2Fcpnp%2Ffiles%2Fabout%2Fuk-ofcom-nutrient-profile-model.pdf

8. Department of Health. *Nutrient Profiling Technical Guidance*. United Kingdom Department of Health; 2011. Accessed June 12, 2022. https://docs.google.com/viewer?url=https%3A%2F%2Fassets.publishing.service.gov.uk%2Fgovernment%2Fuploads%2Fsystem%2Fuploads%2Fattachment_data%2Ffile%2F216094%2Fdh_123492.pdf

9. Grummon AH, Musicus AA, Salvia MG, Thorndike AN, Rimm EB. Impact of health, environmental, and animal welfare messages discouraging red meat consumption: An online randomized experiment. *J Acad Nutr Diet*. Published online October 9, 2022. doi:10.1016/j.jand.2022.10.007

10. Bollard T, Maubach N, Walker N, Mhurchu CN. Effects of plain packaging, warning labels, and taxes on young people's predicted sugar-sweetened beverage preferences: An experimental study. *Int J Behav Nutr Phys Act*. 2016;13(1):95.

11. Vargas-Meza J, Jáuregui A, Contreras-Manzano A, Nieto C, Barquera S. Acceptability and understanding of front-of-pack nutritional labels: An experimental study in Mexican consumers. *BMC Public Health*. 2019;19(1):1751. doi:10.1186/s12889-019-8108-z


© 2025 Grummon AH et al. *JAMA Network Open*

12. Acton RB, Hammond D. Do Consumers Think Front-of-Package “High in” Warnings are Harsh or Reduce their Control? A Test of Food Industry Concerns. *Obesity*. 2018;26(11):1687-1691. doi:10.1002/oby.22311

13. Haws KL, Winterich KP, Naylor RW. Seeing the world through GREEN-tinted glasses: Green consumption values and responses to environmentally friendly products. *Journal of Consumer Psychology*. 2014;24(3):336-354. doi:10.1016/j.jcps.2013.11.002

14. United States Census Bureau. 2023 PUMS Data. Census.gov. September 19, 2024. Accessed December 16, 2024. https://www.census.gov/programs-surveys/acs/microdata/access/2023.html

15. United State Census Bureau. *Income in the United States: 2023*. United Census Bureau; 2024. Accessed December 16, 2024. https://www.census.gov/library/publications/2024/demo/p60-282.html

© 2025 Grummon AH et al. *JAMA Network Open*
