# Supplementary material for: Ecolabels and the Healthfulness and Carbon Footprint of Restaurant Meal Selections: A Randomized Clinical Trial
Source: JAMA Netw Open. 2025 Aug 4;8(8):e2524773. doi: 10.1001/jamanetworkopen.2025.24773 (PMC12322791; doi:10.1001/jamanetworkopen.2025.24773)
Supplement: Supplement 3. — Data Sharing Statement [file jamanetwopen-e2524773-s003.pdf]

Data Sharing Statement

Grummon. Ecolabels and the Healthfulness and Carbon Footprint of Restaurant Meal Selections. *JAMA Netw Open*. Published August 04, 2025. doi:10.1001/jamanetworkopen.2025.24773

Data
Additional Information: ClinicalTrials.gov, NCT#06584539
Data available: Yes
Data types: Deidentified participant data
How to access data: We will post the de-identified data to a public GitHub repository upon publication.
When available: With publication

Supporting Documents
Document types: Statistical/analytic code
How to access documents: We will post the analytic code to a public GitHub repository at https://github.com/clee321/JAMANetOp-foodsgogreen upon publication.
When available: With publication

Additional Information
Who can access the data: Data will be publicly available to anyone who wishes to download them.
Types of analyses: Data will be publicly available for any purpose.
Mechanisms of data availability: Data will be publicly available to anyone who wishes to download them.
Any additional restrictions: NA
